# Bacterial TLR2/6 Ligands Block Ciliogenesis, Derepress Hedgehog Signaling, and Expand the Neocortex

**DOI:** 10.1128/mbio.00510-23

**Published:** 2023-04-13

**Authors:** Beth Mann, Jeremy Chase Crawford, Kavya Reddy, Josi Lott, Yong Ha Youn, Geli Gao, Cliff Guy, Ching-Heng Chou, Daniel Darnell, Sanchit Trivedi, Perrine Bomme, Allister J. Loughran, Paul G. Thomas, Young-Goo Han, Elaine I. Tuomanen

**Affiliations:** a Department of Infectious Diseases, St. Jude Children’s Research Hospital, Memphis, Tennessee, USA; b Department of Immunology, St. Jude Children’s Research Hospital, Memphis, Tennessee, USA; c Department of Developmental Neurobiology, St. Jude Children’s Research Hospital, Memphis, Tennessee, USA; d Hartwell Center for Bioinformatics and Biotechnology, St. Jude Children’s Research Hospital, Memphis, Tennessee, USA; School of Veterinary Medicine, University of Pennsylvania; Harvard Medical School

**Keywords:** cell wall, hedgehog signaling, neocortex, Toll-like receptors

## Abstract

Microbial components have a range of direct effects on the fetal brain. However, little is known about the cellular targets and molecular mechanisms that mediate these effects. Neural progenitor cells (NPCs) control the size and architecture of the brain and understanding the mechanisms regulating NPCs is crucial to understanding brain developmental disorders. We identify ventricular radial glia (vRG), the primary NPC, as the target of bacterial cell wall (BCW) generated during the antibiotic treatment of maternal pneumonia. BCW enhanced proliferative potential of vRGs by shortening the cell cycle and increasing self-renewal. Expanded vRGs propagated to increase neuronal output in all cortical layers. Remarkably, Toll-like receptor 2 (TLR2), which recognizes BCW, localized at the base of primary cilia in vRGs and the BCW-TLR2 interaction suppressed ciliogenesis leading to derepression of Hedgehog (HH) signaling and expansion of vRGs. We also show that TLR6 is an essential partner of TLR2 in this process. Surprisingly, TLR6 alone was required to set the number of cortical neurons under healthy conditions. These findings suggest that an endogenous signal from TLRs suppresses cortical expansion during normal development of the neocortex and that BCW antagonizes that signal through the TLR2/cilia/HH signaling axis changing brain structure and function.

## INTRODUCTION

Microbial components have a range of effects on the brain, the breadth of which is incompletely understood. For instance, bacterial metabolites released from the microbiome are believed to program responses in the brain (1–3). Bacterial structural components such as bacterial cell wall (BCW), a polymerized carbohydrate and amino acid network (4), both stimulate brain inflammation during meningitis (5) and direct noninflammatory responses when released from the gut microbiome to circulate in serum (6–10). Such signals are believed to contribute to neurodegeneration and behavioral abnormalities (11, 12). In the context of the maternal/fetal interface, bacterial metabolites from the microbiome can affect fetal development (1). However, little is known about how bacterial components released by antibacterial treatment of infection during pregnancy affect fetal brain development despite ample evidence that prenatal maternal bacterial infections increase risks for neurocognitive deficits (13–15). One proposed mechanism is the maternal immune activation (MIA), a syndrome whereby inflammation in the mother incites cytokines that relay an inflammatory signal across the placenta to immune cells in the fetus (16, 17). However, this does not address events early in gestation before the fetal immune response is mature or the possibility of a direct interaction of microbial products with Toll-like receptors (TLRs) in fetal neural progenitor cells (NPCs). Along these lines, BCW, the major component of bacterial lytic debris, is recognized by TLR2 (5) present on NPCs even in the early stages of brain development when immune cells are not yet formed (18, 19). We have previously shown that purified BCW can cross the placenta and traffic to the fetal brain, leading to an abnormal increase in cortical neuronal number in a TLR2-dependent fashion (20). Thus, microbial ligands appear to induce events affecting early fetal brain development independent of classical inflammation. Here, we investigate the cellular targets and molecular mechanisms by which BCW expands cortical neuronal numbers.

The primary NPCs are ventricular radial glia (vRGs; also called apical radial glia) that reside in the ventricular zone (VZ) lining the brain ventricle (21–24). To populate the fetal cortex, vRGs can divide symmetrically to increase their pool or asymmetrically to produce one vRG (preserving their pool) and one progeny that differentiates into a neuron, an intermediate progenitor cell (IPC), or an outer radial glia (oRG; also called basal radial glia). IPCs and oRGs form the subventricular zone (SVZ), where they divide to produce neurons (22, 25–29). Young neurons migrate along the radial fibers of RGs to form the six-layered neocortex in an inside-out manner: late-born neurons migrate past early-born neurons to form a more superficial layer closer to the brain surface. Therefore, NPCs control the size and architecture of the brain and understanding the mechanisms regulating NPCs is crucial to understanding brain developmental disorders.

Using a model of pneumococcal pneumonia in pregnant mice followed by antibiotic treatment that floods the fetus with BCW while curing the mother, we describe that vRGs are the cellular target of BCW in the fetal brain. In a restricted window of vulnerability, BCW enhanced proliferative potential of vRGs, resulting in an expanded NPC pool that propagated through the entire period of neurogenesis, increasing neurons in all layers in the neocortex. Remarkably, TLR2 localized at the base of cilia in vRGs and the BCW-TLR2 interaction suppressed ciliogenesis, leading to derepression of Hedgehog (HH) signaling. This noninflammatory, noncanonical TLR2 signaling axis through HH reshaped brain architecture. We also show that TLR6 is an essential partner of TLR2 in this process. Further, TLR6 alone was required to set the normal number of cortical neurons, suggesting there exists an endogenous ligand that is antagonized by BCW.

## RESULTS

### BCW exposure during a restricted developmental window increases neurons in all cortical layers.

**FIG 1:**
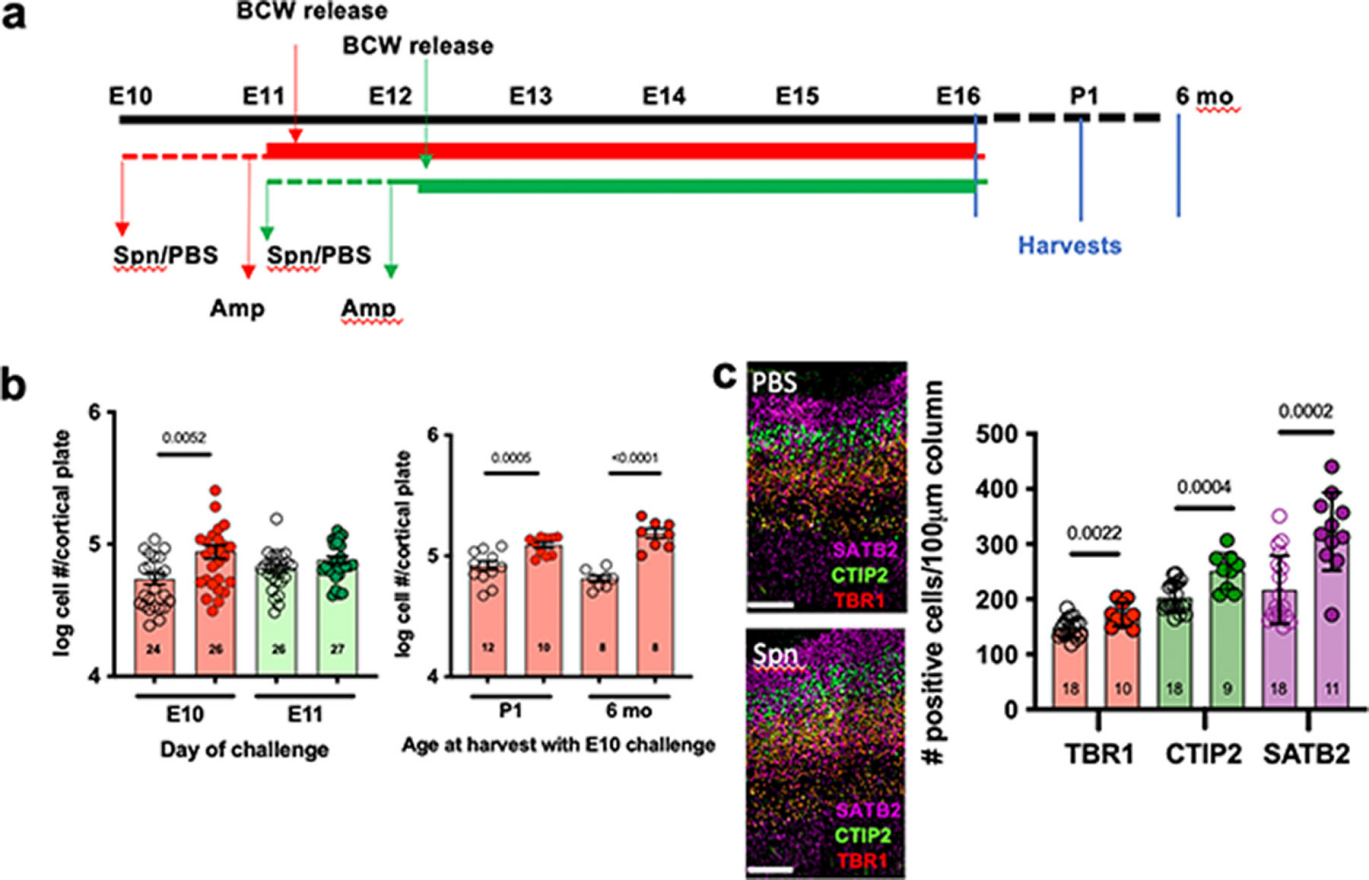
Determining the window of vulnerability for aberrant increase of neurons. (a) Diagram of the experimental design. Dams were challenged with S. pneumoniae or PBS (control) at E10 (red dashed line) or E11 (green dashed line), followed 24 h later by ampicillin (Amp; solid lines) until E16 in all groups. The period of antibiotic-induced BCW release and entry into fetal brain begins 6 h after Amp in the *S. pneumoniae* group (indicated by arrows) (20). The PBS+Amp group controls for any effects of Amp on endogenous maternal microbiota. Fetal brains were collected at times as indicated. (b, left) *S. pneumoniae* challenge on the indicated day and harvest at E16. Right: challenge on E10 and harvest at 1 day or 6 months after birth. Total number of neurons in the entire cortical plate obtained from Nissl-stained sections analyzed by stereology. We measured both hemispheres of the cortex for three to five dams with two to eight embryos/dam for each experimental group: Amp alone (open circles) versus *S. pneumoniae* + Amp (filled circles). Each symbol represents average of three sections per embryo. The number of embryos is indicated in each bar. *P* values were determined by using unpaired, two-tailed *t* tests. No significant effect on size of the cortical plate was noted. (c) E10 challenge, E16 harvest. Sagittal sections. Neurons were stained for TBR1 (layer VI), CTIP2 (layer V), or SATB2 (layer II-IV). Scale bar, 100 μm. Quantification of neurons in each layer in a 100-μm-wide cortical column from the base of the VZ to the top of the cortical plate (see methods): Amp (open circles) versus *S. pneumoniae* + Amp (filled circles). Each symbol averages two sections from one embryo, and each bar represents the means ± the standard errors of the mean (SEM). The number of embryos is indicated in each bar. Spn, *S. pneumoniae*.

Embryonic day 10 (E10) marks the initiation of neurogenesis in the mouse neocortex (23, 24, 30). We previously showed that injecting mothers with purified BCW at E10, but not E15, increases the number of cortical neurons at E16 and P10 (20). To align this observation with a clinically relevant scenario and further narrow the window of vulnerability before E15, we initiated maternal pneumonia by challenge with Streptococcus pneumoniae at E10 or E11, followed at 24 h by treatment with ampicillin (Amp), which generates a rapid release of BCW into the maternal bloodstream. BCW crosses the placenta and is detectable in the fetal brain from 6 h onward (i.e., at E11 or E12, respectively) (Fig. 1a) (20). Treatment of the phosphate-buffered saline (PBS) group with Amp controlled for any antibiotic effects on the endogenous maternal microbiome. An increase in the number of fetal cortical neurons was seen after E10, but not E11, challenge, narrowing the period of vulnerability to direct effects of BCW on fetal neurons to before E11 (Fig. 1a and b).

To define which layers of excitatory neurons were increased by BCW challenge, we labeled neurons with specific layer markers: TBR1 for layer VI, which arises early in development; CTIP2 (also known as BCL11B) for layer V, which arises next; and SATB2 for layers II to IV, which arise last in development (31–33). Bacterial challenge did not affect the layering of the cortex (Fig. 1c). However, the E10 challenge, but not E11, significantly increased the number of neurons in each layer (Fig. 1c; see also Fig. S1 in the supplemental material) even though most SATB2^+^ II-IV neurons arise at E14 to E17 and most TBR1^+^ layer VI neurons arise at E11 to E13. The abnormal cortical architecture persisted after birth (Fig. 1b; see also Fig. S1b). Since BCW is cleared from the fetal brain within 2 to 3 days (20), these findings suggest that an encounter to bacterial products at the onset of neurogenesis establishes a persistent dysregulation long after the exposure.

10.1128/mbio.00510-23.1 FIG S1 Determining the window of vulnerability for aberrant increase of neurons. Dams were challenged at E10 or E11 as shown in Fig. 1a and harvested at E16 or P1. (a) Neuronal layers were stained for TBR1 (red, layer VI), CTIP2 (green, layer V), or SATB2 (purple, layers II to IV). Scale bar, 100 μm. (b) Quantification of neurons in each layer in a 100 μm wide cortical column from the base of the VZ to the top of the cortical plate: Amp (open circles) versus *S. pneumoniae* + Amp (filled circles). We measured at least three dams per condition with two to seven embryos/dam. Each symbol averages two sections from one embryo, and each bar represents the means ± the SEM. The number of embryos is indicated in each bar. *P* values were determined by unpaired two-tailed *t* tests. Spn, *S. pneumoniae*. Download FIG S1, TIF file, 2.4 MB. Copyright © 2023 Mann et al. 2023 Mann et al. https://creativecommons.org/licenses/by/4.0/ This content is distributed under the terms of the Creative Commons Attribution 4.0 International license.

Several lines of evidence distinguished the fetal NPC response from MIA. Expansion of the neocortex did not correlate with maternal serum cytokine levels since these were elevated after both E10 and E11 challenges (see Fig. S2a), despite fetal vulnerability only after E10 challenge. The response also occurred before substantial appearance of fetal microglia as indicated by equivalent, low levels of the microglial activation marker IBA1 at both E10 and E11 (see Fig. S2b). These features argued against MIA.

10.1128/mbio.00510-23.2 FIG S2 Absence of inflammatory activity in fetal brain despite maternal cytokine production. (a) Maternal serum cytokine response was measured at 24 h after *S. pneumoniae* challenge at E10 or E11. There were two to five dams per group. NS, *P > *0.05. (b) Stain for microglial activation marker IBA1 (red) and nucleus (blue DAPI) in fetal brains 48 h after challenge at E10 or E11. Scale bar, 100 μm. Spn, *S. pneumoniae*. Download FIG S2, TIF file, 4.8 MB. Copyright © 2023 Mann et al. 2023 Mann et al. https://creativecommons.org/licenses/by/4.0/ This content is distributed under the terms of the Creative Commons Attribution 4.0 International license.

It has been suggested that fetal exposure to microbial products from maternal infection is linked to permanent disorders of behavior or neuropsychiatric diseases (13–15). Consistent with this, bacterium-challenged mice showed spatial recognition defects, repetitive behaviors, and sociability defects (see Fig. S3), indicating that abnormal brain development may lead to abnormal architecture and disordered postnatal behavior in humans who experienced maternal bacterial infection during early fetal development.

10.1128/mbio.00510-23.3 FIG S3 Behavioral consequences of aberrant increase of neurons. (a) Spatial recognition test using a Y maze. Briefly, mice were exposed to two of three arms in a Y-maze for 8 min with the third arm gated. One hour after returning to its cage, the mouse was allowed to explore all three arms for 5 min. Time and entries were noted for all arms. There was no sexual dimorphism in any behavior model. We compared numbers of entries into a novel arm by 2-, 4-, and 6-month-old mice exposed at E10 to either Amp (blue bars) or *S. pneumoniae* + Amp (red bars). No differences were noted at 2 and 4 months (data not shown), while at 6 months of age, bacterium-challenged mice were less likely to enter a novel arm of the Y-maze and for longer periods of time, while control mice explored both original and novel arms equally. *P* values were determined by two-way ANOVA with Bonferroni’s Multiple comparison test. (b) We tested offspring at 2, 4, and 6 months of age for repetitive behavior (time spent grooming) and sociability (time spent at a transparent, perforated partition upon the addition of a nonlittermate mouse). To assess repetitive behavior, mice were placed alone in a new cage and video recorded for 1 h. The time spent grooming was tracked by a human observer. Control mice significantly decreased the amount of time they spent self-grooming with age, while all the bacterium-challenged mice continued to show high levels of repetitive behavior of self-grooming. To study sociability, a white box divided by a perforated Plexiglas was used. Experimental mice were acclimated to one side of the box for 8 min with the other side empty. The mouse was returned to its cage for 1 h and then subsequently, placed back in the box with a nonlittermate mouse on the other side of the divide. The number of approaches to the divider as well as time spent attempting to interact with the nonlittermate was calculated. Values represent means ± standard deviation of two to three assays, four to six mice per assay. *P* values were determined by ANOVA. In a sociability assay at 6 months of age, the challenged group interacted significantly less with a companion mouse than the control group. Spn, *S. pneumoniae*. Download FIG S3, TIF file, 2.0 MB. Copyright © 2023 Mann et al. 2023 Mann et al. https://creativecommons.org/licenses/by/4.0/ This content is distributed under the terms of the Creative Commons Attribution 4.0 International license.

### Rapid initial expansion of vRGs by shortening the cell cycle time.

**FIG 2:**
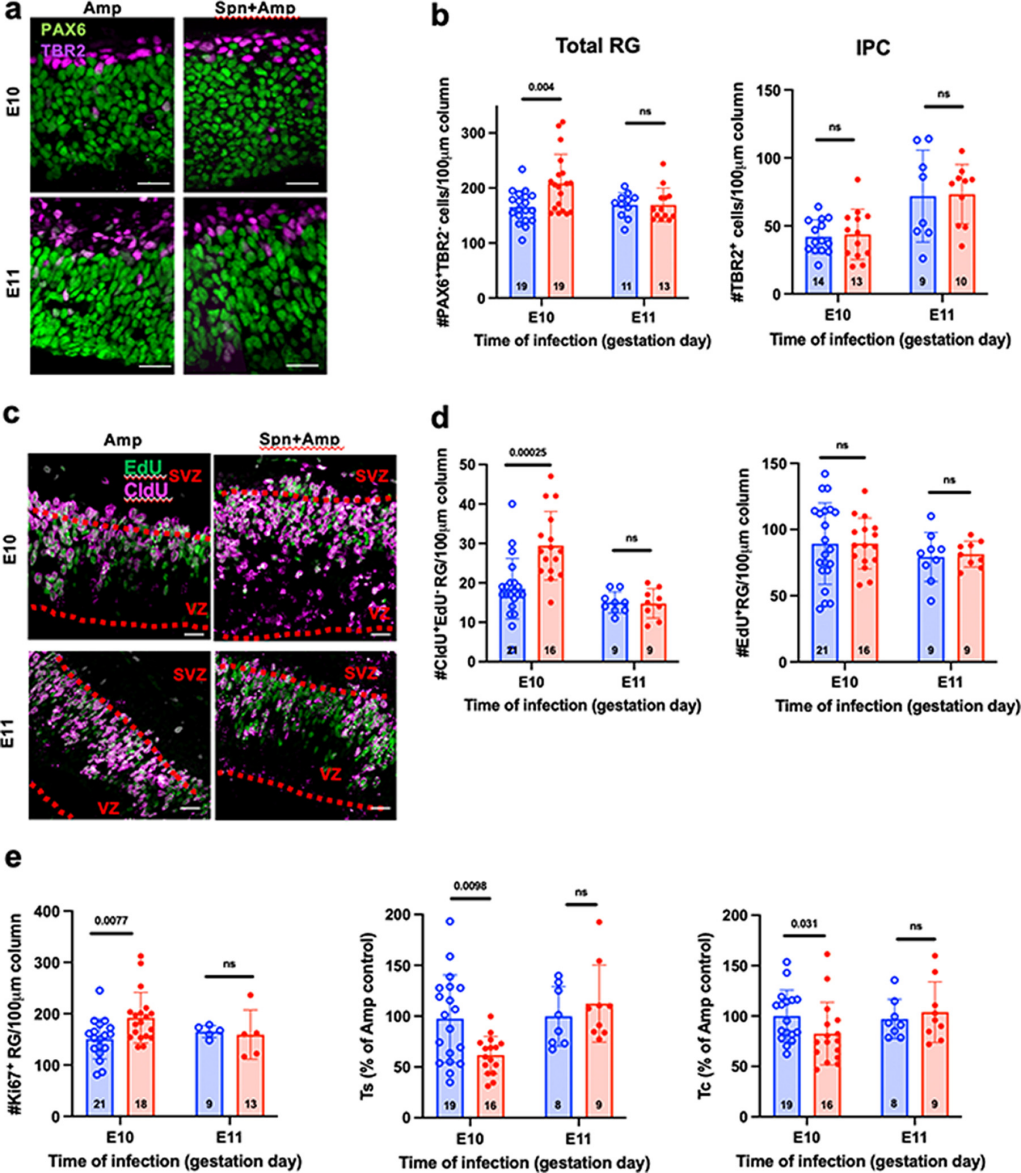
*S. pneumoniae* challenge expands vRGs and shortens vRG cell cycle. (a and b) *S. pneumoniae* challenge at E10 but not E11 expands vRGs. We challenged dams at E10 or E11 (blue, PBS; red, *S. pneumoniae*), treated them with Amp 24 h later, and quantified RGs (PAX6^+^ TBR2^–^ cells; green) and IPCs (PAX6^–^ TBR2^+^ cells; purple) 48 h later (E12 or E13, respectively). Two sections were quantified from each embryo, and bars represent the means ± the SEM of the number of embryos indicated in each bar. (c to e) Cell cycle kinetics of RGs in the 48 h after challenge at E10 or E11. Blue, Amp; red, *S. pneumoniae* + Amp. We injected CldU and EdU at 2 and 0.5 h before harvest, respectively. Note the many CldU^+^ EdU^–^ cells in the lower VZ (c) in embryos that were challenged with bacteria at E10 and treated with Amp. The graphs show RGs that had left S phase (CldU^+^ EdU^–^; panel d, left), total RGs in S phase (EdU^+^; panel d, right), RGs in cell cycle (Ki67^+^; panel e, left), and the relative lengths of S phase and the cell cycle of RGs (panel e, middle and right; control Amp alone set at 100%). The data are shown as means ± the SEM. *P* values were determined by unpaired two-tailed *t* tests. Spn, *S. pneumoniae*.

vRGs are the primary NPCs that produce neurons, either directly or through oRGs and IPCs. We hypothesized that an initial expansion of vRGs followed by increased production of oRGs and IPCs could create a progressive wave of increased neurons into all layers of the cortical plate. Consistent with our hypothesis, vRGs (PAX6^+^ TBR2^–^ cells in the VZ) but not IPCs (TBR2^+^ cells) were greatly expanded by 1 day after antibiotic treatment with E10, but not E11, challenge (Fig. 2a and b), indicating that vRGs amplified themselves as primary targets of BCW.

We next investigated how BCW caused vRGs to expand. The division mode of NPCs is closely associated with cell cycle kinetics (34). In particular, vRGs undergoing self-amplifying divisions show a shorter cell cycle than vRGs producing differentiating progenies (35). Thus, we investigated if the expanded vRGs were characterized by a shortened cell cycle by using a double thymidine analogue labeling method with successive injections of 5-chloro-2′-deoxyuridine (CldU) at 2 h (h) and 5-ethynyl-2′-deoxyuridine (EdU) at 0.5 h before harvest (36, 37). EdU^+^ PAX6^+^ TBR2^–^ cells represent vRGs in S phase at the time of harvest, whereas CldU^+^ EdU^–^ cells represent vRGs that have left S phase and entered G_2_ phase during the 1.5 h between CldU and EdU injections (Fig. 2c). The nuclei of vRGs show interkinetic migration (37): nuclei undergo S phase at the upper part of the VZ, move down to the ventricular surface during G_2_ phase, and divide at the ventricular surface. Consistent with interkinetic nuclear migration of vRGs, the nuclei of vRGs in S phase (EdU^+^) were concentrated in the upper part of the VZ (Fig. 2c). Remarkably, many CldU^+^ EdU^–^ vRG nuclei were present in the lower VZ in BCW-challenged but not control E10 embryos, suggesting that more cells exited S phase and entered G_2_ phase during the 1.5 h interval in E10-challenged than control embryos (Fig. 2c). Accordingly, E10 challenge, but not E11, dramatically increased the number of CldU^+^ EdU^–^ cells compared to control (Fig. 2d, left) indicating an increased exit from S phase. However, the total number of vRGs in S phase (EdU^+^ cells) did not change (Fig. 2d, right), indicating that as exit from S phase increased so did the number of vRGs entering S phase. Although the number of vRGs increased after E10 challenge (Fig. 1b), the number of vRGs in S phase (EdU^+^ vRGs) did not increase, indicating the proportion of vRGs in S phase was decreased in E10 challenged embryos. Since the length of a cell cycle is proportional to the number of the cells in that cell cycle these findings are indicative of a shortened S phase. Indeed, challenge at E10, but not E11, significantly shortened the lengths of S phase and the cell cycle (Fig. 2e) calculated according to the method of Nowakowski et al. (see Materials and Methods) (37). Together, these results show that E10 *S. pneumoniae* challenge expanded vRGs by shortening the cell cycle length.

### Initial expansion of vRGs leads to expanded NPCs in later stages.

**FIG 3:**
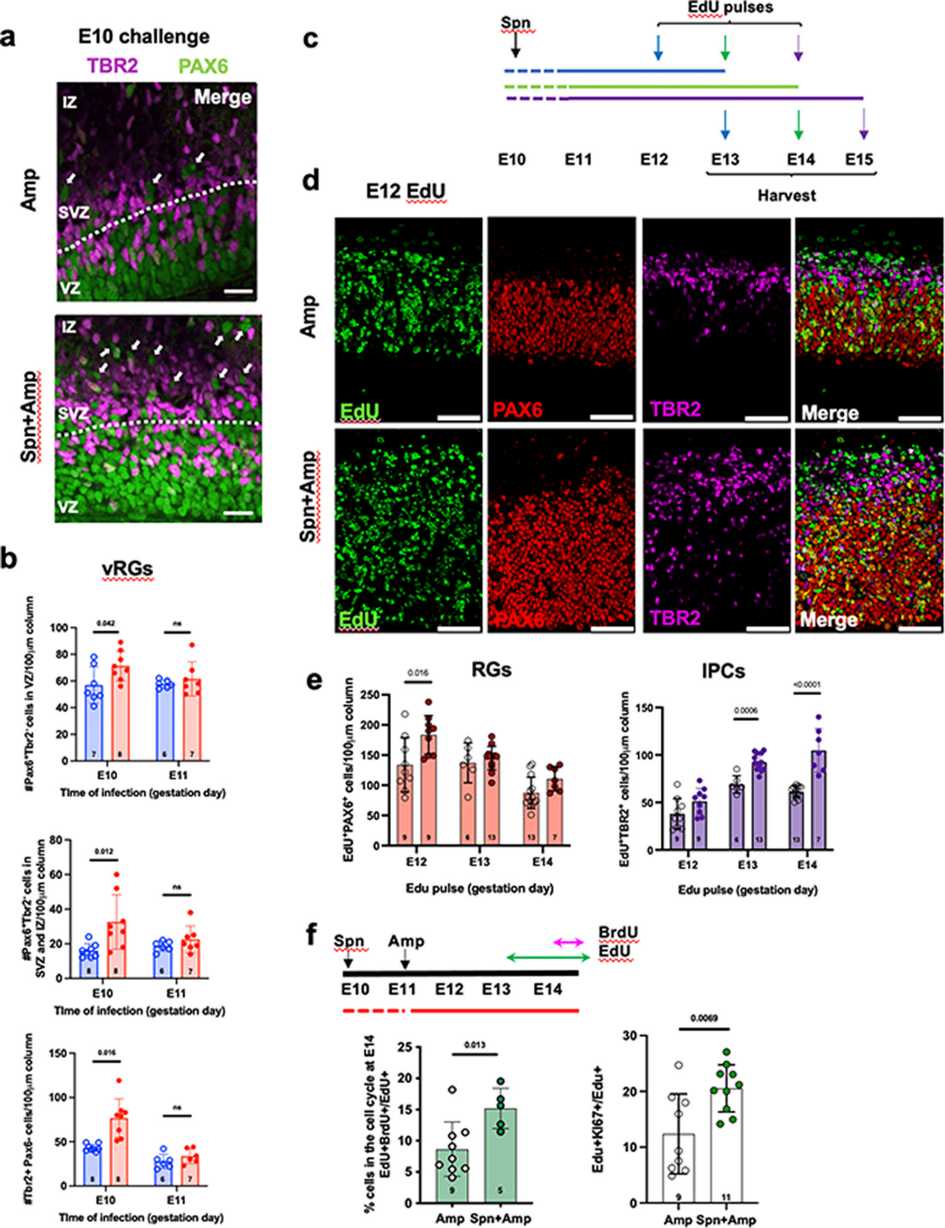
Propagation of expanded vRGs into oRGs and IPCs. (a and b) Expanded vRGs increased numbers of NPCs later. We challenged dams with bacteria at E10 or E11 and quantified NPCs at E16 by PAX6 and TBR2 markers as well as location. vRGs: PAX6^+^ TBR2^–^ cells in the VZ (regions defined by white dotted line). oRGs: PAX6^+^ TBR2^–^ cells outside the VZ. IPCs: TBR2^+^ cells in the SVZ. Blue, Amp; red, *S. pneumoniae* + Amp. oRGs were further defined as PAX6^+^ TBR2^–^ cells that also expressed HOPX (see Fig. S9). Arrows indicate examples of oRGs. (c to e) Proliferation of NPCs in later developmental stages after the E10 challenge. (c) We administered *S. pneumoniae* at E10, Amp at E11, and EdU at E12, E13, or E14, and harvested brains 24 h later. (d) E13 brain sections stained for PAX6, TBR2, and EdU. Scale bar, 100 μm. (e) Numbers of EdU^+^ RGs and IPCs in challenged (filled circles) versus control (open circles) brains. (f) NPC self-renewal was assessed by labeling with EdU at E13 (green arrow), followed by a BrdU pulse (pink arrow) at 1.5 h before harvest at E14. Self-renewed cells (EdU^+^ BrdU^+^) were expressed as the percentage of total EdU^+^ cells (left). The samples were also stained for the proliferation marker Ki67 as a function of EdU^+^ (right). *P* value was determined by an unpaired two-tailed *t* test. Spn, *S. pneumoniae*.

The increase in neuron number in all cortical layers suggests that the early expansion of vRGs was maintained and propagated into downstream cell lineages throughout the neurogenic period. To test this possibility, we quantified the number of vRGs, oRGs, and IPCs (as defined in Fig. 3) at E16, a late stage of cortical neurogenesis. All 3 cell types were increased solely in the E10 challenged group, consistent with a time-restricted vulnerability of vRGs to BCW (Fig. 3a and b). The increase of oRGs and IPCs indicates that the initial expansion of vRGs resulted in the subsequent expansion of downstream NPCs. These results suggest that E10 challenge expanded all NPC types by E16.

10.1128/mbio.00510-23.9 FIG S9 Identification of oRGs by HOPX immunofluorescence stain. Representative images of WT brain sections challenged at E10 with *S. pneumoniae *+ Amp or Amp and harvested at E16. Images show the VZ identified by radial glial marker PAX6 (green) and the SVZ/IZ identified by IPC marker TBR2 (purple). HOPX (red), which was shown to be enriched in oRGs in fetal human brain (70), was expressed in both vRGs and oRGs in mice consistent with the previous reports that HOPX is expressed in both vRGs and oRGs in human cerebral organoids, ferrets, and mice (40, 71, 72). PAX6^+^ TBR2^–^ HOPX^+^ cells in the SVZ are denoted by white arrows indicative of oRGs in challenged animals. Spn, *S. pneumoniae*. Download FIG S9, TIF file, 5.9 MB. Copyright © 2023 Mann et al. 2023 Mann et al. https://creativecommons.org/licenses/by/4.0/ This content is distributed under the terms of the Creative Commons Attribution 4.0 International license.

To understand how expanded vRGs increased NPCs in later developmental stages, we labeled proliferating NPCs by pulses of EdU at E12, E13, or E14 and quantified EdU^+^ cells in the VZ and SVZ 24 h later (Fig. 3c and d). EdU injection labeled more RGs and IPCs at all stages in the challenged group than in control with the highest increase of EdU^+^ vRGs at E12 while EdU^+^ IPCs showed strong increases at E13 and E14 injection times (Fig. 3e). These results suggest that more vRGs expanded at E12 in the challenged group than in control and that increased vRGs subsequently produced more IPCs at E13 and E14. This supports a model that E10 challenge resulted in a wave of expansion of both RGs and IPCs over time in their expected developmental sequence, with vRGs impacted first, followed by IPCs.

Next, we determined whether the expanded NPCs were sustained into later stages through self-renewal. We injected dams with EdU at E13 and with BrdU at 1.5 h before harvest at E14 (Fig. 3f) to identify progenitors that proliferated at E13 (EdU^+^), remained as progenitors, and proliferated again at E14 (BrdU^+^). The proportion of self-renewed progenitors (EdU^+^ BrdU^+^) was significantly increased in E10 challenged group. Consistently, more EdU^+^ cells were positive for Ki67, a proliferation marker. These findings showed that the initial expansion of vRGs at E12 propagated to the expansion of all three NPC types at later stages in E10 challenged embryos through increased proliferative capacity and self-renewal.

### BCW components require TLR2/6 to expand neurons.

**FIG 4:**
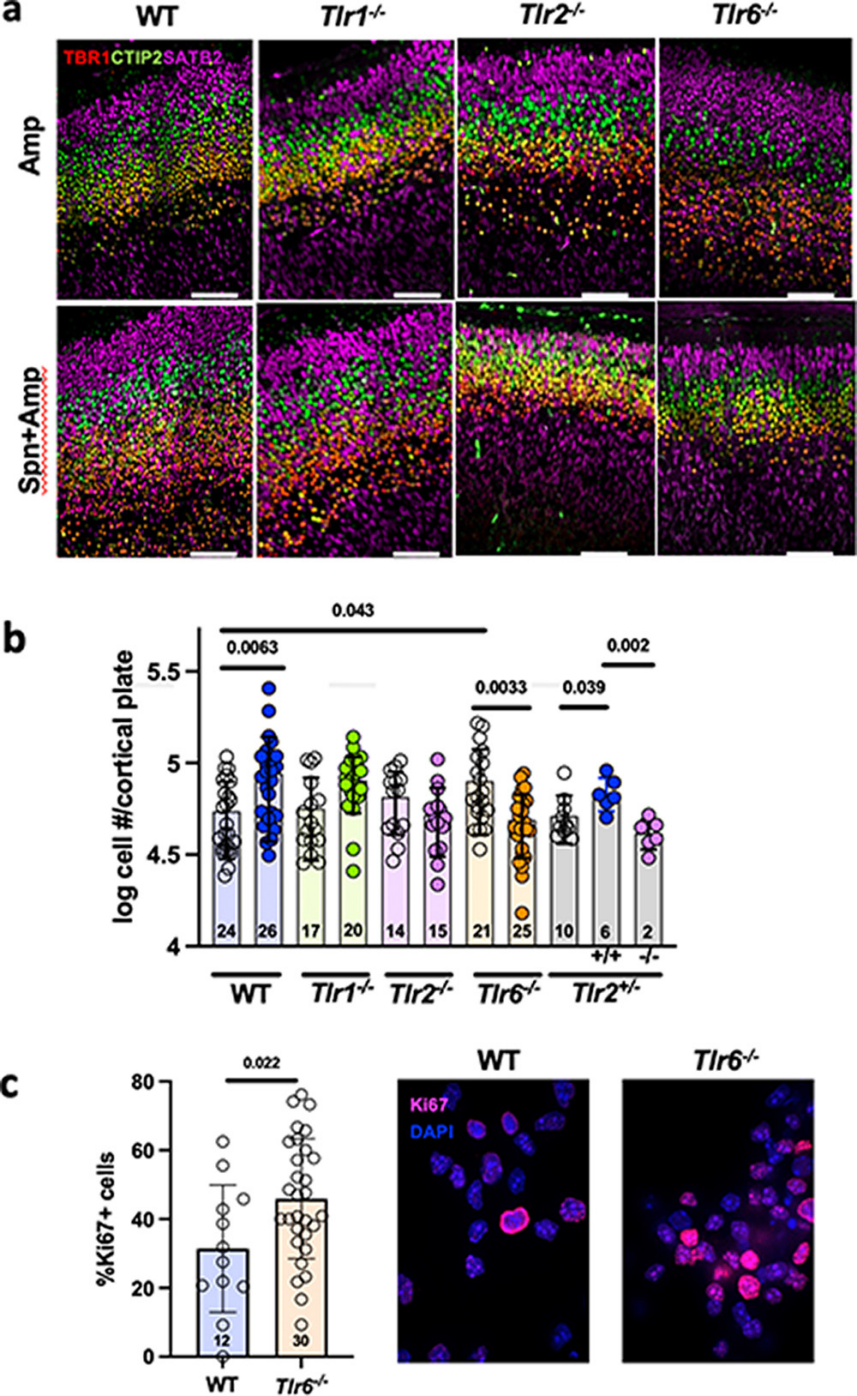
*S. pneumoniae* challenge requires TLR2/6 to expand cortical neurons. (a and b) Effect of *Tlr1*, *-2*, or -*6* deficiency on cortical expansion. Dams deficient in *Tlr1*, *-2*, or -*6* were challenged with *S. pneumoniae* or PBS at E10, treated with Amp at E11, and harvested at E16. In addition, heterozygous *Tlr2^+/–^* parents were crossed, mothers were challenged as described above and embryonic brains were analyzed in accordance with fetal genotype (gray bars). TBR1, CTIP2, and SATB2 staining shows normal layering. Scale bar, 100 μm. Graphs show the total numbers of neurons in the entire cortical plate from Nissl-stained sections analyzed by stereology: Amp (open circles) versus *S. pneumoniae* + Amp (filled circles). Each symbol represents the average of three sections per embryo (the number of embryos is indicated in each bar). *P* values were determined by a two-way ANOVA with Tukey’s multiple-comparison test. (c) NPCs from WT or *Tlr6^–/–^* cells were cultured for 5 days and stained for proliferation marker Ki67 (pink) and DAPI (blue) and quantified. Spn, *S. pneumoniae*.

BCW interacts with TLR2 (5). TLR2 is expressed by both NPCs and embryonic neurons and TLR2 expression is maintained at a similar level during the challenge period (18, 19). The present study identified NPCs (vRGs) as the specific target of BCW with subsequent impact on all types of cortical neurons. TLR2 functions as a heterodimer with either TLR1 or TLR6. The impact of either TLR2 signaling combination in fetal brain development or NPC proliferation is unknown. To identify which TLR2 heterodimer mediates BCW effects in NPCs, we challenged pregnant mice bearing wild-type (WT), *Tlr1^−/−^*, *Tlr2^−/−^*, or *Tlr6^−/−^* embryos with *S. pneumoniae* or PBS at E10, treated with Amp, and examined the brains at E16. Neuronal layering was normal in all mutant embryos (Fig. 4a). However, *S. pneumoniae* challenge increased the number of cells in the cortical plate in WT and *Tlr1^−/−^* embryos but not in *Tlr2^−/−^* and *Tlr6^−/−^* embryos (Fig. 4b), demonstrating that the TLR2/6 axis is required for BCW to increase cortical neurogenesis. This result could be ascribed to the genotype of the fetus, and not the mother, as shown by the comparison of *Tlr2^−/−^* versus *Tlr2^+/–^* fetuses carried by *Tlr2^+/–^* mothers. Neurons increased in *Tlr2^+/–^* but not *Tlr2^−/−^* fetal brains (Fig. 4b, gray bars).

### Loss of TLR signaling affects normal brain architecture.

We next tested whether loss of TLR signaling affected brain structure in unchallenged controls. Remarkably, the number of cortical neurons was significantly greater in the untreated *Tlr6^−/−^* embryos compared with WT (Fig. 4b). TUNEL staining showed few, if any, apoptotic cells, in both WT and *Tlr6^−/−^* untreated brain sections, ruling out that less apoptosis in *Tlr6^−/−^* brains accounted for increased neurons (see Fig. S4). On the other hand, more untreated *Tlr*6^−/−^ NPCs cultured *in vitro* expressed a proliferation marker, Ki67, than untreated WT NPCs (Fig. 4c), suggesting that augmented NPC proliferation underlies increased production of neurons. Further work will be necessary to reveal how endogenous TLR6 signaling limits the number of cortical neurons in healthy WT animals. Nonetheless, the increase in the baseline neuronal number in unchallenged *Tlr6^−/−^* mice establishes the requirement for an unknown endogenous TLR6 axis signal to set the number of neurons during normal neurodevelopment and that TLR ligands, such as BCW, modulate that signal. Together, our results indicate that TLR6 suppresses cortical neuronal expansion under healthy conditions and that pathogenic TLR ligands reverse such suppression.

10.1128/mbio.00510-23.4 FIG S4 Absence of apoptosis in untreated WT and *Tlr6^–/–^* fetal brains. Representative sections of fetal cortex at E16 were stained by TUNEL (terminal deoxynucleotidyltransferase-mediated dUTP-biotin nick end labeling) to assess prevalence of apoptosis as a function of fetal genotype. Few if any apoptotic cells were present in sections, while a positive-control section shows strong staining (brown). Spn, *S. pneumoniae*. Download FIG S4, TIF file, 1.5 MB. Copyright © 2023 Mann et al. 2023 Mann et al. https://creativecommons.org/licenses/by/4.0/ This content is distributed under the terms of the Creative Commons Attribution 4.0 International license.

### TLR2/6 signaling derepresses Hedgehog signaling.

**FIG 5:**
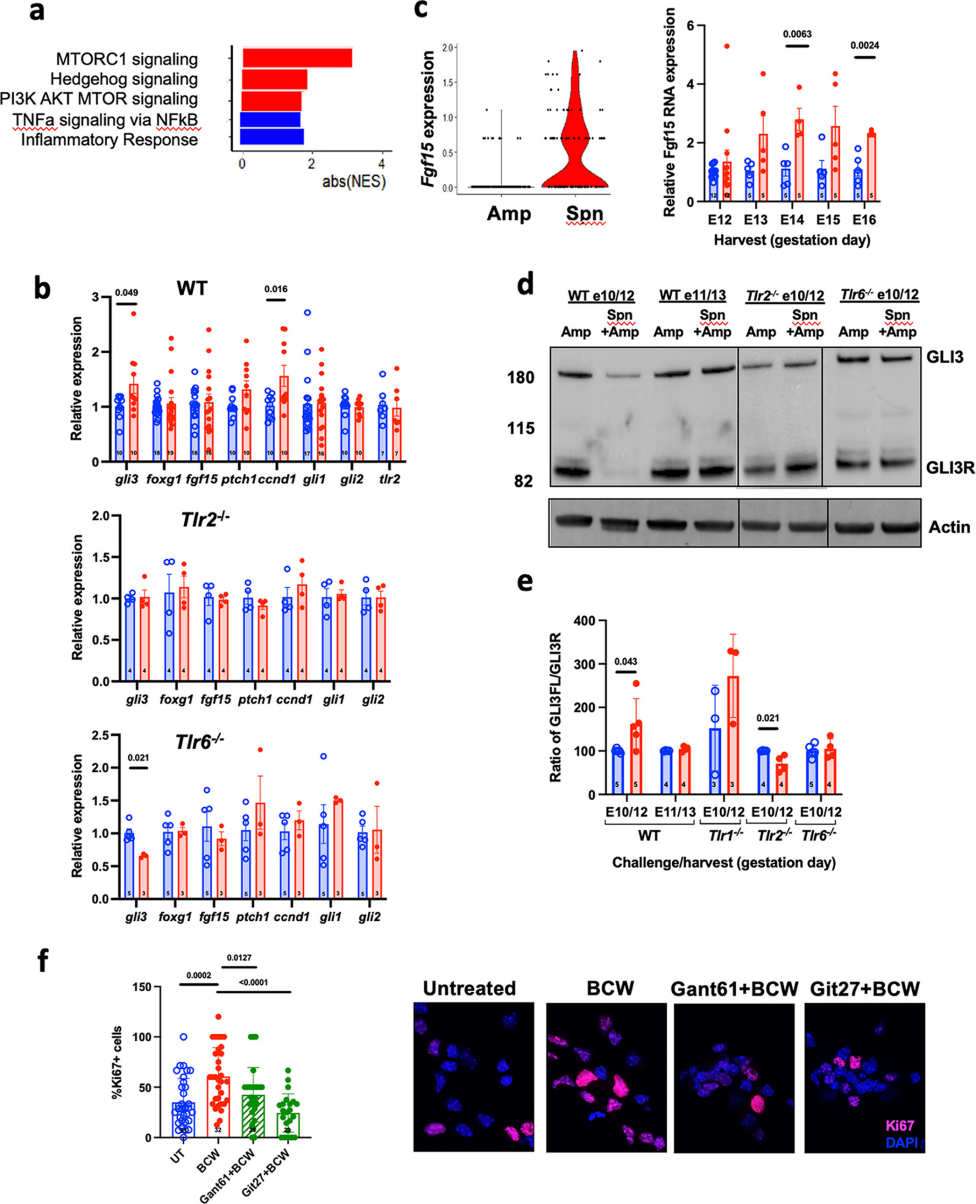
TLR2/6 signaling derepresses HH signaling. (a) Preranked GSEA was performed comparing control to *S. pneumoniae*-challenged E12 VZ gene expression assessed by spatial transcriptomics. Absolute values of the normalized enrichment scores (NES) for pathways of interest that met statistical significance (FDR < 0.05) are presented. (b) Relative expression of the indicated genes as assessed by RT-qPCR in brain lysates of WT or *Tlr2^−/−^* or *Tlr6*^−/−^ embryos exposed to Amp (blue) or *S. pneumoniae* + Amp challenge (red). (c, left) Violin plot of *Fgf15* expression obtained from control (Amp) and treated (*S. pneumoniae* + Amp) E12 VZ as assessed by spatial transcriptomics. (Right) *Fgf15* expression levels compared by RT-qPCR. Challenge occurred at E10 and samples were harvested at the indicated day. Values represent three to four dams and three to five embryo lysates per dam. The data are shown as means ± the SEM. *P* values were determined by an unpaired two-tailed *t* test. (d and e) WT, *Tlr2^−/−^*, and *Tlr6^−/−^* dams were challenged at the indicated day with PBS (blue) or *S. pneumoniae* (red) and treated with Amp 24 h later, and brains were harvested after another 24 h, as indicated. Brain lysates were assessed by Western blotting for full-length GLI3 activator form (GLI3FL) and GLI3R (processed repressor) (d) (Fig. S8). (e) Ratio of protein levels of GLI3FL to GLI3R. We used two to three dams per condition with two to four embryos/dam. *P* values for all panels were determined by unpaired, two-tailed *t* tests. Gels were spliced for labeling purposes and to remove unwanted unnecessary background. (f) NPCs harvested at E11 were plated *in vitro* and pretreated with or without inhibitors of GLI (GANT61, 5 μM) or TLR2/6 (Git27, 10 μg/mL) for 2 h, followed by BCW (MOI of 0.1) in the presence of inhibitor. Proliferation was assessed by Ki67^+^ staining. Spn, *S. pneumoniae*.

To understand the molecular mechanism for BCW-induced expansion of vRGs, we used spatial transcriptomics to compare gene expression in E12 brains from dams challenged at E10 with *S. pneumoniae* or PBS and treated at E11 with Amp. Comparisons focused on the VZ, where vRGs reside and are marked by expression of PAX6. Several signaling pathways, including HH, mTOR, and PI3K/AKT, were significantly enriched within the *S. pneumoniae*-challenged VZ, while tumor necrosis factor and the inflammatory response was not (Fig. 5a; see also Fig. S5 and Table S1). Enrichment of HH and PI3K/AKT/MTORC1 signaling was notable because both induce the expansion of NPCs and the growth of the neocortex (38–42), which were the consequences of BCW challenge at E10. Activation of PI3K/AKT/MTORC1 signaling *in vitro* and in the brains of challenged mice depended on TLR2 (see Fig. S6). Reverse transcription-quantitative PCR (RT-qPCR) confirmed that multiple genes known to be upregulated by activation of HH signaling in the embryonic neocortex (43, 44) were upregulated upon BCW challenge but not in *Tlr2^−/−^* dams (Fig. 5b). The levels of *Gli1*, the well-known readout of strong HH signaling were not upregulated. This result was in fact consistent with the previous finding that the loss of GLI3, which suppresses the expression of HH target genes, increases the expression of HH target genes but not *Gli1* in the developing neocortex at this stage in the neocortex (44). This suggests that *Gli1* expression may require much stronger activation of HH signaling than that achieved by GLI3 loss or *S. pneumoniae*. On the other hand, *Fgf15*, a target of HH signaling in the developing cortex, which is upregulated by GLI3 loss and expands vRGs by shortening their cell cycle (44), was upregulated in the E10 challenged group and remained upregulated between E12 and E16 (Fig. 5c).

10.1128/mbio.00510-23.5 FIG S5 Impact of *S. pneumoniae* challenge on VZ gene expression analyzed by spatial transcriptomics. Dot plot visualizing expression patterns across selected genes of interest, particularly genes known to be downstream of HH signaling. Dot size corresponds to the proportion of capture areas expressing a gene. Color corresponds to the scaled average expression. VZ, ventricular zone. The full list of significantly DEGs obtained by comparing E12 control to E12-treated ventricular zone regions can be accessed in Table S1. Spn, *S. pneumoniae*. Download FIG S5, TIF file, 0.9 MB. Copyright © 2023 Mann et al. 2023 Mann et al. https://creativecommons.org/licenses/by/4.0/ This content is distributed under the terms of the Creative Commons Attribution 4.0 International license.

10.1128/mbio.00510-23.6 FIG S6 Effect of inhibition or loss of TLRs on PI3K/AKT pathway activity after BCW/*S. pneumoniae* challenge. (a) Effect of TLR antagonists on NPC pAKT and PI3K protein levels *in vitro*. NPCs were cultured *in vitro* from E11 embryos and treated with PBS (blue bar) or BCW (red bar). Git27 (TLR2/6 antagonist) or CU-CPT22 (TLR 2/1 antagonist) were treated 2 h before BCW treatment. Cells were harvested 2 h later. Protein levels are expressed as a percentage of those in vehicle-treated cells. Each bar represents the means ± the SEM of four independent experiments. (b) Effect of *Tlr* deficiency on pAKT and PI3K protein levels *in vivo*. WT and *Tlr2^−/−^* dams were challenged at the indicated day with PBS (blue) or *S. pneumoniae* (red) and treated with Amp 24 h later, and brains were harvested another 24 h later, as indicated. Brain lysates were assessed by Western blotting for p-AKT, AKT, or PI3K levels. Values are expressed as a percentage of WT control. *P* values for all panels were determined by unpaired, two-tailed *t* tests. Spn, *S. pneumoniae*. Download FIG S6, TIF file, 1.7 MB. Copyright © 2023 Mann et al. 2023 Mann et al. https://creativecommons.org/licenses/by/4.0/ This content is distributed under the terms of the Creative Commons Attribution 4.0 International license.

10.1128/mbio.00510-23.10 TABLE S1 Significantly regulated genes by *S. pneumoniae* challenge. Spatial transcriptomic analysis at E12 of significantly regulated genes comparing *S. pneumoniae* challenge at E10 versus control. See Materials and Methods for statistical analysis parameters. Spn, *S. pneumoniae*. Download Table S1, EPS file, 0.6 MB. Copyright © 2023 Mann et al. 2023 Mann et al. https://creativecommons.org/licenses/by/4.0/ This content is distributed under the terms of the Creative Commons Attribution 4.0 International license.

10.1128/mbio.00510-23.8 FIG S8 GLI3 protein assessed by Western blotting. Western blots that were used to quantify the ratio of GLI3FL to GLI3R protein from individual embryonic brain lysates after *S. pneumoniae* challenge at E10 or E11 and harvested 48 h later. Each set represents samples from two to three independent experiments. Spn, *S. pneumoniae*. Download FIG S8, TIF file, 2.1 MB. Copyright © 2023 Mann et al. 2023 Mann et al. https://creativecommons.org/licenses/by/4.0/ This content is distributed under the terms of the Creative Commons Attribution 4.0 International license.

To understand how BCW enhanced HH signaling, we examined GLI3, a transcription factor whose activity is directly regulated by HH signaling (44, 45). In the absence of active HH signaling, full-length GLI3 (GLI3FL) is proteolytically cleaved to become GLI3 repressor (GLI3R). GLI3R suppresses the expression of HH target genes in vRGs and its loss strongly increases the expression of HH target genes, including *Fgf15* (44). Consistent with the enrichment of HH signaling signatures, including increased *Fgf15*, the GLI3FL activator levels relative to GLI3R levels were significantly increased in E10-challenged fetal brains in a TLR2 and TLR6 dependent manner (Fig. 5d and e). Remarkably, treatment with a GLI inhibitor (GANT61), as well as a TLR2/6 inhibitor (Git27), significantly blocked BCW-induced proliferation of NPCs *in vitro* (Fig. 5f). These findings collectively suggest that BCW acting through TLR2/6 expands NPCs at least in part by increasing the ratio of GLI3 activator to repressor, resulting in the derepression of HH target gene expression.

### BCW suppresses ciliogenesis via TLR2/6.

**FIG 6:**
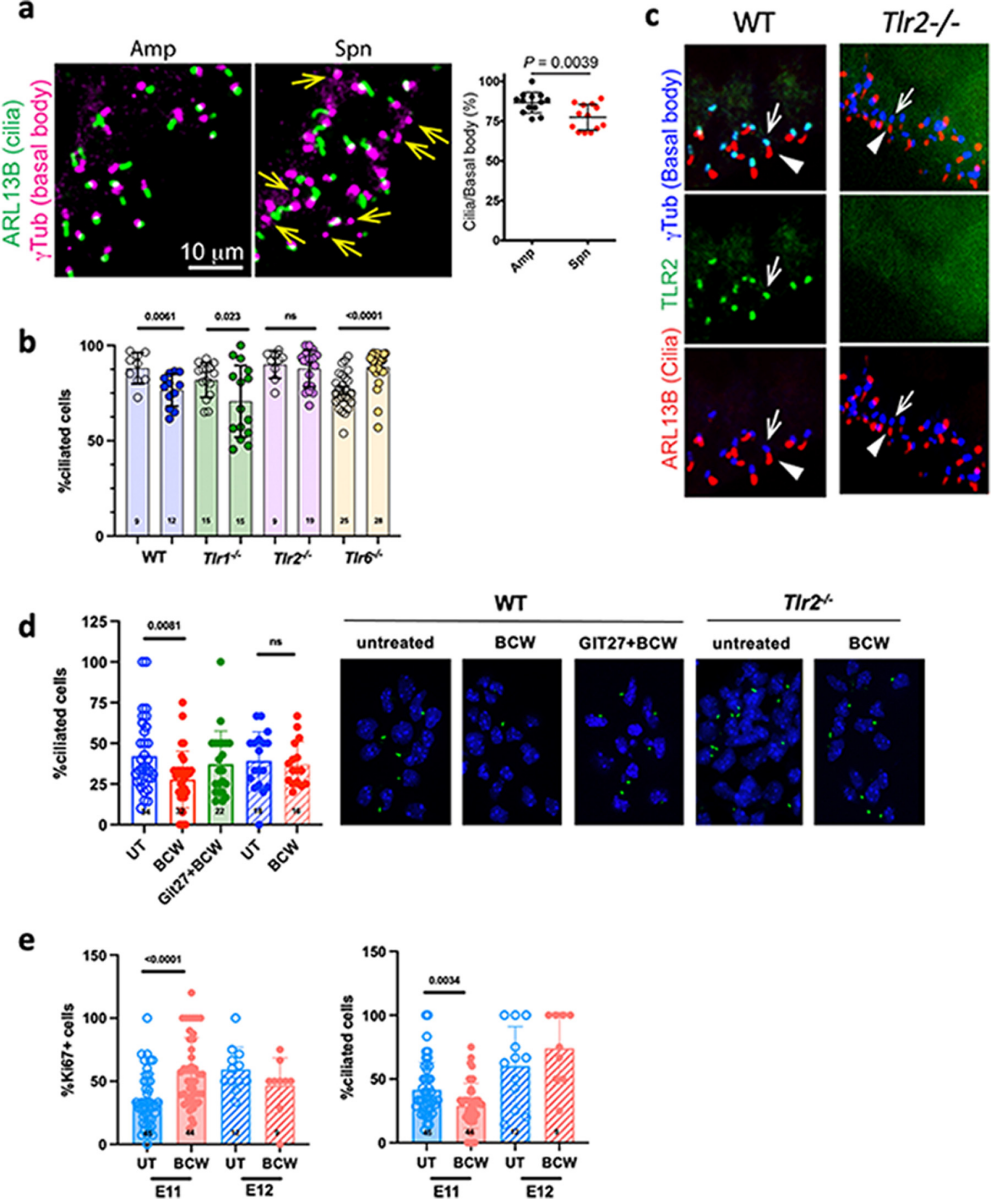
*S. pneumoniae* challenge suppresses ciliogenesis via TLR2. (a) *S. pneumoniae* challenge decreases the number of primary cilia. Dams were challenged at E10 with PBS or *S. pneumoniae* and treated with Amp 24 h later, and brains were harvested at E12. Pictures show the E12 cortex stained for basal body (γTub, magenta) and primary cilia (ARL13B, green). Arrows point to examples of basal bodies lacking primary cilia in the *S. pneumoniae*-challenged group. The graph shows the percent basal bodies associated with primary cilia (means ± the SEM) quantified from 10 sections from three embryos per group. The *P* value was determined by unpaired two-tailed *t* test. (b) Quantification of basal bodies bearing primary cilia as a function of *Tlr* genotype. WT and *Tlr^−/−^* dams were challenged at E10 as indicated, and fetal brains were harvested at E12, prepared, and analyzed as described for panel a. Amp (open circles) versus *S. pneumoniae* + Amp (filled circles) results are indicated. (c) Localization of TLR2 to base of cilia. Pictures of E12 VZ of WT (control) or *Tlr2^−/−^* pups stained for γTub (basal body marker, blue), ARL13B (primary cilia marker, red), and TLR2 (green) are shown. The bottom row shows the basal body (arrows) is located at the base of cilia (arrowheads); the middle row shows TLR2 (green); and at the top is a merge of the middle and bottom images showing colocalization (cyan) of TLR2 with γTub at the base of cilia in vRGs. TLR2 staining is absent in sections from *Tlr2^−/−^* mice. Scale bar, 10 μm. The experiment was performed in duplicate in two pups per group. (d) WT (solid bars) and *Tlr2^−/−^* (hatched bars) NPCs were harvested at E11, cultured *in vitro* for 24 h, washed, and pretreated with or without Git27 (10 μg/mL) for 2 h, followed by BCW (MOI of 0.1) for 24 h. The percentage of cells bearing cilia was quantified by fluorescence imaging (cilia, ARL13B, green; nucleus, DAPI, blue). Values are the average of three experiments. The *P* value was determined by an unpaired two-tailed *t* test. (e) WT NPCs were harvested at E11 (solid bars) or E12 (hatched bars), cultured *in vitro* with or without BCW (MOI of 0.1) for 24 h. Cells were stained for proliferation (Ki67^+^) or cilia by fluorescence imaging (cilia, ARL13B, green; nucleus, DAPI, blue). Values are averages of three experiments. The *P* value was determined by an unpaired two-tailed *t* test. Spn, *S. pneumoniae*.

GLI3FL must shuttle through primary cilia to be proteolytically cleaved to become GLI3R (46, 47). Accordingly, the loss of cilia in NPCs leads to the loss of GLI3R and derepression of HH target genes, including *Fgf15* (44). Remarkably, BCW challenge at E10 significantly decreased the number of vRGs with primary cilia (Fig. 6a). Moreover, this effect was lost in *Tlr2^−/−^* or *Tlr6^−/−^* mice (Fig. 6b). TLR2 localized to primary cilia in vRGs (Fig. 6c). To further test whether BCW decreased cilia through TLR2/6, we treated NPC cultures with BCW in the presence or absence of TLR2/6 inhibitor Git27 (Fig. 6d). Consistent with *in vivo* observations, BCW decreased the number of NPCs with cilia. This decrease was blocked by the TLR2/6 inhibitor Git27. Moreover, BCW failed to decrease cilia in NPCs lacking TLR2. Also consistent with *in vivo* observations, BCW altered cilia and proliferation of NPCs cultured from E11 but not E12 embryos (Fig. 6e). Together, these findings suggest that BCW-TLR2/6 signaling from cilia suppresses the formation of cilia and GLI3R, leading to the derepression of HH target genes and the expansion of NPCs.

## DISCUSSION

The maternal fetal interface monitors a constant, undercurrent dialog between the fetus and the mother, including responses to microbial metabolites released from the maternal microbiota or inflammatory molecules that characterize MIA (1–3). Here, we investigate the cellular targets and molecular mechanisms of effects of microbial products that cross the placenta and interact directly with fetal cortical NPCs. MIA is known to affect fetal brain development (16, 17). Several lines of evidence argue against this as a mechanism for the NPC expansion observed by BCW challenge. NPC expansion occurred at E10 but not E11 challenge despite similar maternal serum cytokine profiles at both time points. Further, the fetal TLR genotype determined the NPC expansion response regardless of the TLR genotype of the mother, which may affect MIA. Finally, microglia, the target of MIA, only begin to appear at E10 and thus challenged brains would have very few if any microglia. Even so, E11 would have a higher number, and yet no response was seen, arguing against microglial function in explaining the E10 response.

The present study examined the consequences of the release of bacterial lytic products in the maternal bloodstream in a context relevant to clinical maternal/fetal care as initiated by β-lactam antibiotic therapy to cure pregnant mice with pneumococcal pneumonia. In this model, a burst of BCW release occurs over 6 to 24 h after antibiotic administration as bacteria rapidly die, creating a pulse of bacterial debris that accumulates in the fetal brain. *S. pneumoniae* challenge at E10 (but not E11), followed by Amp treatment to create BCW, increased the number of all excitatory neuronal types of the neocortex, indicating a wide impact of bacterial components released in the mother on fetal brain architecture. These structural changes were accompanied by cognitive and behavioral abnormalities indicating that bacterial infection and treatment in the early stages of pregnancy could have long-lasting effects on brain structure and function.

BCW increased the number of excitatory neurons in all cortical layers although they arise at different times spanning E11.5 to E17.5 (48). An effect initiated at E10, but spanning many days thereafter, suggested that the consequences of BCW challenge originated in the pool of NPCs and were reflected in all cells born afterward. Indeed, all NPCs (vRGs, oRGs, and IPCs) underwent expansion. We suggest that vRGs are the primary target of BCW because only PAX6^+^ TBR2^–^ NPCs were expanded initially and immediately after the challenge. It is unclear why only vRGs respond to BCW. BCW might be present in cerebrospinal fluid, to which only vRGs are exposed. vRG expansion later progressed as a wave in time through the generation of later cell types.

Before producing neurons, vRGs divide symmetrically to amplify themselves. As they switch to divide asymmetrically to produce neurons, the cell cycle lengthens (35, 49). Our data suggest that signals from bacterial debris such as BCW expand the initial pool of vRGs at the early stage of cortical development by delaying the lengthening of their cell cycle and increasing self-amplifying divisions. This initial expansion of vRGs was propagated to the expanded pool of IPCs and oRGs that were also maintained by increased self-renewal. It is notable that BCW expanded both IPCs and oRGs, a feature thought to underlie the development and evolution of the large and folded neocortex in higher mammals, including humans (24, 50–53). This emphasizes that bacterial components could have profound effects on features of human cortical development.

Activation of TLRs in the postnatal brain by pathogens is well-known to induce an innate immune response. During bacterial meningitis, BCW interacts with TLR2/1 to initiate inflammation and neuronal damage (5, 54, 55). TLRs are expressed in the fetus as early as E10; however, it has remained unclear whether BCW/TLRs might act as morphogens in mammalian neurodevelopment independent of inflammation, as seen in *Drosophila* (56, 57). Our data show that activation of TLR2 and TLR6, but not TLR1, increased fetal neurogenesis and altered brain morphology in the absence of inflammation. Thus, by switching partners in the heterodimer at different time points of development, TLR2 participates in either fetal neurogenesis (TLR2/6) or postnatal inflammation (TLR2/1) in mammals.

In addition to the participation of TLRs in responses to BCW in challenged fetuses, unchallenged *Tlr6^−/−^* mice showed a significantly increased neuronal number compared to unchallenged WT mice. Fittingly, TLR6 loss increased the number of proliferating NPCs. This suggests a requirement for an endogenous TLR6 axis signal to limit the number of neurons during normal neurodevelopment and that TLR ligands, such as BCW, modulate that signal. Together, our results might indicate that TLR2 and TLR6 suppress cortical neuronal expansion under healthy conditions and that pathogenic TLR ligands reverse such suppression. Further studies will be necessary to test this hypothesis.

Spatial transcriptomics of control versus challenged fetal brains revealed an enrichment in genes associated with several signaling pathways. PI3K/AKT signaling was enhanced consistent with our previous findings that it promotes NPC proliferation downstream of TLR2 *in vitro* (20). In contrast, HH signaling has not been previously connected to TLRs. Our data suggest that BCW activated HH signaling indirectly via suppressing cilia and GLI3R formation. Supporting this, a previous study showed that TLR2 promotes the disassembly of cilia in cultured cells (58). We revealed that TLR2 localized to cilia in vRGs and that BCW challenge decreased the number of vRGs with cilia in a TLR2/6-dependent manner *in vitro* and *in vivo*. Thus, TLR2 appears to signal to disassemble the primary cilium. Since vRGs form the wall of the cerebral ventricle and project cilia into the cerebrospinal fluid, the association of TLRs with cilia may promote their function as sentinels. Moreover, as shown for the genetic ablation of cilia in vRGs at early stages of corticogenesis (44), the decrease in ciliated vRGs in BCW-challenged embryos was accompanied by increased activator to repressor ratio of GLI3 and derepression of HH target genes, including *Fgf15*, which shortens the cell cycle and promotes proliferation of vRGs. Importantly, genetic removal of cilia or GLI3 by E12.5 but not by E14.5 expands NPCs and the cortex (43, 44, 59). Notably, E10 to E12 is a critical developmental window when vRGs switch from symmetric self-amplifying divisions to asymmetric neurogenic divisions (60). This switch in division modes is critical to determining the number of neurons and the size of the cortex because it determines the number of founding vRGs that subsequently generate all the excitatory neurons in the neocortex directly or through secondary progenitors (IPCs and oRGs). *S. pneumoniae* challenge at E10 and ampicillin treatment at E11 released BCW during E11 to E12, exactly overlapping with the time when vRGs switch division modes. Importantly, challenge at E10 induced changes similar to the loss of GLI3 by E12, while challenge at E11 did not because the release of BCW occurred after the critical period when vRGs switch division modes. These findings highlight the importance of developmental timing in NPC responses to regulatory factors and provide a possible explanation for the time-dependent effects of *S. pneumoniae* challenges.

We, therefore, propose a novel neurodevelopmental pathway that is driven by direct targeting of NPCs by BCW and connects TLRs to morphogenesis. TLR2, newly found at the base of primary cilia of vRGs, modulates the HH/GLI3/FGF15 pathway to expand NPCs leading to permanent changes in the trajectory of development of neocortical architecture and neurocognitive function. This process is independent of TLR2/1-induced inflammation and neuronal death that characterizes BCW-induced responses from the innate immune system after birth. Importantly, in *Tlr6^−/−^* control embryos without BCW challenge, the loss of TLR6 increased the number of cortical neurons, suggesting that an endogenous TLR signal regulates normal neurodevelopment in the embryo and that BCW released from maternal infection antagonizes that signal.

An important next question is whether BCW affects the development of interneurons and the ratio of inhibitory interneurons to excitatory neurons. Does BCW affect the proliferation of interneuron progenitors in the ventral telencephalon? If BCW selectively expands excitatory neurons, does that result in a lower-than-normal density of interneurons or an increased survival of interneurons and normal density of interneurons in response to expanded excitatory neurons? Of note, HH signaling plays critical roles in specifying and maintaining molecularly distinct NPC types that produce diverse classes of interneurons (61, 62). Does BCW alter interneuron fates by affecting HH signaling in interneuron progenitors? A lower-than-normal density of interneurons, perhaps with differential effects on interneuron classes, might result in excitatory/inhibitory imbalance contributing to neurocognitive deficits associated with prenatal maternal bacterial infections in humans and in our mouse model.

## MATERIALS AND METHODS

### *S. pneumoniae* live infection maternal challenge model.

Wild-type C57BL/6 and *Tlr1^−/−^* mice were obtained from The Jackson Laboratory; C57BL/6 *Tlr6^−/−^* were obtained from Oriental Bioservices, Inc.; C57BL/6 *Tlr2^−/−^* (5) were bred in-house. Table 1 contains genotyping primer sequences. All experiments were carried out in accordance with institutional and NIH guidelines.

**TABLE 1:** Primers used to verify gene knockouts for mice in this study

Strain	Vendor	Primer	Sequence (5′–3′)
*Tlr1 KO* (B6.129S1_*Tlr1*)	Jackson	oIMR6966	GCCAAACGCAAACCTTACCAGAGTG
		oIMR6967	ACGGACACATCCAGAAGAAAACGG
		oIMR6968	TTCGGCTATGACTGGGCACAACAG
		oIMR6969	TACTTTCTCGGCAGGAGCAAGGTG
*Tlr2* KO	SJCRH	PM.STJ.583	CTTCCTGAATTTGTCCAGTACA
		PM.STJ.584	GGGCCAGCTCATTCCTCCCAC
		PM.STJ.585	ACGAGCAAGATCAACAGGAGA
*Tlr6* KO	Oriental Bioservices, Inc.	Wild	GAAATGTAAATGAGCTTGGGGATGGCG
		Extra	TAATCAGAACTCACCAGAGGTCCAACC
		Neo	ATCGCCTTCTATCGCCTTCTTGACGAG

Timed pregnancies were dated as follows: mating pairs were together for 24 h and then separated. Dams were checked for vaginal plugs at 48 h and were palpated every other day. When positive by palpation (ca. E6 to E8), mice were anesthetized and scanned using 40 MHz center frequency transducer (MX550D) following each uterine horn to count the number of embryos.

Dams were challenged intratracheally at E10 or E11 with either PBS (controls) or 10^6^ CFU S. pneumoniae strain TIGR4X in PBS. Twenty-four hours after challenge, blood titers were obtained to ensure consistent bacterial load (mean, 10^5^ CFU/mL; for continued inclusion in the study challenged dams required ≥10^4^ CFU/mL and controls required no colonies at detection threshold of 10^3^ CFU/mL), and the dams were treated with ampicillin (100 mg/kg) intraperitoneally twice daily until embryos were harvested for analysis. A control of challenge alone with no antibiotic treatment was not performed as the mice would succumb to infection.

For some experiments, bromodeoxyuridine (BrdU; 50 μg/g, Sigma, B5002), 5-chloro-2′-deoxyuridine (CldU; 43 μg/g; Sigma, C6891), and/or 5-ethynyl-2′-deoxyuridine (EdU; 10 μg/g; Invitrogen, A10044) were injected intraperitoneally into the pregnant dams at designated times before euthanasia for harvest of embryos.

### Neural progenitor cell culture.

Embryonic brains were harvested between E11 and E13 as indicated, and the cortical hemispheres were separated from the ganglionic eminences and the meninges. The tissue was processed using the Brainbits protocol, and cells were plated at a density of 400,000 cells/well on poly-D-lysine-coated plates or coverslips in complete neurobasal media. At 24 h after seeding, the cells were washed and incubated for 24 h with purified BCW (at a multiplicity of infection [MOI] of 0.1, prepared as described previously [20]). In some cases, cells were pretreated for 2 h prior to BCW challenge with TLR2/6 antagonist Git27 (10 μg/mL), TLR2/1 antagonist Cu-CPT22 (1 μM), or GLI inhibitor GANT61 (5 μM). For analysis, cells were either lysed as described for Western blotting or imaged for proliferation by Ki67 staining or presence of cilia (cilia, ARL13B, green; nucleus, DAPI, blue).

### Immunohistochemistry.

**(i) Frozen sections.** Embryonic brains were fixed in 4% paraformaldehyde overnight, cryoprotected in 30% sucrose at 4°C, frozen in M1 embedding matrix (Thermo Scientific, 1310), and cut sagittally to a thickness of 12 μm. For staining, sections were placed in boiling antigen retrieval buffer (10 mM sodium citrate [pH 6.0]) for 10 min, rinsed in PBS, and placed in blocking solution (5% donkey serum, 0.1% Triton X-100 in PBS) for 45 min. Sections were incubated with primary antibodies in blocking solution overnight at 4°C, rinsed three times in PBS, incubated with secondary antibody at room temperature for 2 h, rinsed three times in PBS, stained with of Hoechst dye for 10 min, and mounted in Prolong diamond antifade mounting media (Invitrogen, P36961).

**(ii) Paraffin sections.** Embryonic brains were harvested as described above, fixed in 10% formalin solution, embedded in paraffin, and sectioned as 4-μm-thick sections.

**(iii) Staining procedures.** Nissl (Sigma, C5042), TUNEL (EMD Millipore, S7101), Iba1 (CST 17198), and EdU (Invitrogen, C10632) staining was performed according to the manufacturer’s instructions. For BrdU costaining, primary and secondary antibody treatment was performed first, followed by denaturing in 2 N HCl for 30 min at 37°C. Sections were rinsed in 0.1 M borate solution (pH 8.5) three times for 5 min each time, followed by PBS washes. Primary antibody solution containing anti-BrdU antibody was applied to the sections and placed in 4°C overnight, followed by the fluorescence staining method described above. A Tyramide Super-Boost kit (Invitrogen, B40922) was used for triple staining for TLR2, gamma tubulin, and ARL13B according to the manufacturer’s protocol. The primary and secondary antibodies used for immunohistochemistry are listed in Table 2.

**TABLE 2:** Key antibodies used for immunofluorescence studies

Antibody or stain	Source	Dilution or concn (μg/mL)
Primary antibodies (IF)		
Anti-tbr1	Abcam, ab31940	1:300
Anti-ctip2	Abcam, ab18465	1:300
Anti-satb2	Abcam, ab51502	1:300
Anti-tbr2	eBioscience, 14-4875-82	1:300
Anti-pax6	R&D, AF8150	1:100
Anti-ki67	Abcam, ab15580	1:100
Anti-ph3	CST 9706	1:100
Anti-cldu	NovusBio, NB500-169	1:100
Anti-gamma tubulin	Sigma, T5192	1:1,000
Anti-arl13b	UC Davis NeuroMap	1:2,000
Anti-tlr2	Abcam, ab209216	1:100
Anti-iba1	CST 17198	1:100
Primary antibodies		
Anti-hopx1	Proteintech, 11419-1-AP	1:500
Anti-gli3	R&D, AF3690	1:200
Anti-pi3k	Thermo Fisher Sci, PA5-19833	1:1,000
Anti-phospho-AKT	CST 4060	1:2,000
Anti-AKT	CST 4691	1:1,000
Anti-beta actin	Sigma, A5441	1:20,000
Secondary antibodies		
CF 488A-conjugated donkey anti-rat IgG	Biotium, 20027	1:500
CF 488A-conjugated donkey anti-rabbit IgG	Biotium, 20015	1:500
CF 488A-conjugated donkey anti-mouse IgG	Biotium, 20014	1:500
CF 568-conjugated donkey anti-rabbit IgG	Biotium, 20098	1:500
CF 568-conjugated donkey anti-mouse IgG	Biotium, 20105	1:500
CF 647-conjugated donkey anti-rat IgG	Biotium, 20843	1:500
CF 647-conjugated donkey anti-rabbit IgG	Biotium, 20047	1:500
CF 647-conjugated donkey anti-mouse IgG	Biotium, 20046	1:500
Alexa Fluor 647-conjugated anti-goat IgG	Thermo Fisher Sci, A21447	1:200
Alexa Fluor 488-conjugated anti-mouse IgG	Thermo Fisher Sci, A28175	1:200
Alexa Fluor 568-conjugated anti-rabbit	Thermo Fisher Sci, A11011	1:200
		1:100
Nuclear stains		
DAPI	Sigma, D9542	1
Hoechst 33342	Biotium, 40046	2–5

### Microscopy.

**(i) Stereology.** Both frozen and paraffin-embedded sagittal sections were stained with cresyl violet, and the entire cortical plate was contoured and counted by using StereoInvestigator with a Cavalieri estimator and an optical fractionator probe (MBF Biosciences) (a representative zone is shown in Fig. S7a in the supplemental material). Both right and left sides of the cortex were measured and the average of three sections per embryo was calculated.

10.1128/mbio.00510-23.7 FIG S7 Marking of brain regions used for analysis. (a) Representative E16 sagittal brain slice stained with cresyl violet (low and high magnification). The cortical plate (outlined in black) is the area contoured and counted by stereology. CP, cortical plate; IZ, intermediate zone; SVZ, subventricular zone; VZ, ventricular zone; HC, hippocampus. (b) Location of a column in the VZ and SVZ regions of an E15 brain used for quantification. Columns (box) were drawn at 100 μm in width and extended vertically from the ventricular boundary to the superior aspect of the desired region (VZ, VZ+SVZ, cortical plate). (c) Representative example of VZ marked for spatial transcriptomics. Mapping of the area of the fetal brain for spatial transcriptomic analysis was determined by manual delineation of anatomical markers for VZ as described in Materials and Methods and utilizing spatial transcriptomics to verify that the chosen region expressed VZ markers FOXG1 and PAX6. Spn, *S. pneumoniae*. Download FIG S7, TIF file, 2.5 MB. Copyright © 2023 Mann et al. 2023 Mann et al. https://creativecommons.org/licenses/by/4.0/ This content is distributed under the terms of the Creative Commons Attribution 4.0 International license.

**(ii) Fluorescence image acquisition and analysis.** Images were acquired on a Nikon C2 (Nikon NIS elements software) or a 3i Marianas system (Slidebook software) using a 40× or 63× objective. For frozen sections of 12-μm thickness, a z-stack of 13 optical sections at a step size of 0.34 μm and with a tiling of multiple images were combined for quantification analysis. For paraffin sections, a two-dimensional montage of a tiled image of the cortical plate region of the right hemispheres was captured using a 40× oil objective on the Marianas. Using Imaris ×64 (Bitplane) image analysis software, one to three sagittal sections of the entire cortex from each E16 embryo were counted in a rectangular column of cortex at the level of the choroid plexus roof plate evaginated telencephalic vesicle, as indicated by the arrow in Fig. S7b. For consistent quantification of progenitor cells in slices, the VZ was defined as the area lining the ventricle and containing dense PAX6^+^ TBR2^−^ nuclei up to the area where cells uniformly express TBR2 (RGs and newborn IPs express PAX6; IPs express TBR2); the SVZ as the second cell-dense area containing uniformly TBR2^+^ cells above the VZ and below a cell-sparse area. Continuous stretches of TBR2^+^ cells form the boundary between the VZ and SVZ. We defined oRGs as PAX6^+^ TBR2^−^ cells above the VZ. Counting was done by at least two blinded individuals to ensure unbiased analysis.

**(iii) Cell cycle analysis.** We injected CldU at 2 h before harvest and EdU at 0.5 h before harvest. The vRGs that have left S phase during the 1.5-h interval between the CldU and EdU injections will be marked as CldU^+^ EdU^−^ (L cells = CldU + EdU^−^ Pax6^+^ Tbr2^−^). The ratio of the length of a period of the cell cycle to that of another period is equal to the ratio of the number of cells in each period. Thus, we determined the length of S phase (*Ts*) from the equation 1.5 h/*Ts* = L cells/S cells, where S cells are EdU^+^ Pax6^+^ Tbr2^−^, and then the length of cell cycle (*Tc*) from the equation *Ts*/*Tc* = S cells/proliferating RGs (Ki67^+^ PAX6^+^ TBR2^–^; Ki67 labels all phases of the cell cycle).

### Embryonic brain lysis.

Individual embryonic brain cortices lysed in 250 μL ice-cold RIPA buffer (Sigma, R0278) with protease inhibitors (CST 5872S). The tissue was homogenized and vortexed for 2 h at 4°C. Lysates were centrifuged to collect debris and supernatant was harvested.

### Western blot analysis.

The concentration of total protein in each sample was determined by BCA (Thermo Scientific), and equal amounts were loaded onto a 4 to 12% SDS-PAGE. The antibodies used for Western blot analysis are listed in Table 2.

### RNA isolation and RT-qPCR.

RNA was extracted from embryonic cortices with an RNeasy kit (Qiagen, 74104) and used for cDNA synthesis by using a Superscript III first-strand synthesis kit (Invitrogen, 18080-400). RT-qPCR was performed using a TaqMan Fast Advanced Master Mix (Applied Biosystems, 4444557) for *FoxG1* Mm02059886_s1, *Fgf15* Mm00433278_m1, *Gli1* Mm00494654_m1, *Gli2* Mm01293117_m1, *Gli3* Mm00492337_m1, *Ptch1* Mm00436026_m1, *Ccnd1* Mm00432359_m1, and endogenous control *Gapdh* Mm99999915_g1 (Applied Biosystems). Five to twelve samples were analyzed, and each sample was run in triplicate reactions.

### Behavioral analysis.

Pups born to mothers treated at E10 with *S. pneumoniae* plus ampicillin or with ampicillin alone were assessed for cognitive function and repetitive behaviors at 2, 4, and 6 months of age. Gender equal numbers of pups from at least three litters (*n* > 12/group) per assay were tested. All assays were recorded by video and quantified by a blinded human observer. Working memory, repetitive behavior, and sociability were assessed as previously described (20, 63).

### Spatial transcriptomics.

Data were generated and analyzed under the direction of Jeremy Crawford who was blinded to the experimental groups. Whole heads from E12 embryos were used for analysis with a Visium spatial transcriptomics kit (10X Genomics, PN-1000184). Immediately after dissection, samples were frozen in isopentane chilled by liquid nitrogen, embedded in OCT (Sakura, 4583), and then stored at −80°C. The, 10-μm sagittal sections were obtained beginning at the periphery to the midline according to 10X Genomics-suggested practices. Tissue permeabilization was optimized to 12 min. A single section (representative zone shown in Fig. S7c) was then obtained from each of four mice per condition, and two sections were placed on each Visium slide (i.e., one section from each condition). Sections were imaged on a Nikon Eclipse N*i*-E scope, and slide fiducials and tissue coverage areas were manually annotated using Loupe Browser (10X Genomics). Libraries were prepared according to manufacturer recommendations and sequenced on an Illumina NovaSeq platform with a sequencing configuration of 28-10-10-120 (R1-i7-i5-R2) at >200 million clusters per library. Data were analyzed using SpaceRanger (v1.2.1; 10X Genomics) using the corresponding mm10 reference, with the reverse read trimmed to 90 bp.

Downstream analyses were performed using the STUtility (v0.1.0) (64) and Seurat (v4.0.3) (65) packages in the R environment (v4.1.0), with each library independently processed using SCTransform (regressing out the percentage of mitochondrial expression per spot) and then merged into a single object. Capture areas overlaying putative ventricular zone regions were identified by anatomical recognition based on the prenatal mouse brain atlas (66) and confirmed by the abundance of *foxg1* and *pax6* overlapping expression in these sites, and analyses focused on these regions. Differentially expressed genes (DEGs) between conditions were determined using the Seurat FindMarkers function with default parameters, with *P* values adjusted for multiple comparisons using Bonferroni correction. Gene set enrichment analysis (GSEA) (67) was performed using GSEAPreranked on a DEG list obtained using FindMarkers with min.pct set to 0.01 and logfc.threshold set to 0, with ranks ordered by average log_2_-fold change; for this analysis, Hallmarks (v7.4) (68) gene sets were queried using the Mouse MSigDB symbol remapping chip (v7.0) with a classic enrichment statistic. To ensure that the conclusions drawn from spatial transcriptomics experiments were rigorous, experiments included multiple, biologically independent sample replicates for each point of comparison. All statistical analyses are corrected for type 1 error (false discovery rate) by adjusting *P* values using the Benjamini-Hochberg method (69). All data and accompanying documentation are archived on servers both in the laboratory and in the St. Jude Children’s Research Hospital cloud, which is backed up daily.

### Statistics.

We used Prism version 6.05 (GraphPad) for statistical analysis. Detailed statistical parameters are in each figure legend. All male and female embryos were analyzed. Groups contained at least two to three pregnant mice that yielded at least four to six embryos/dam, a cohort size giving a power of 0.95 to detect a 0.5-log change in cell number at a *P* value of 0.01. The treatment assignment, challenge status, and endpoint analysis were blinded to investigators.

Data analysis was performed to achieve robust and unbiased results. Unless otherwise specified, comparisons of phenotypes were analyzed by calculating the mean and standard deviation. *P* values were calculated using a two-tailed, unpaired Student *t* test for two-group comparisons or using two-tailed *t* test with Welch’s correction or a Mann-Whitney test in Prism version 6.05 (GraphPad). In instances where multiple experimental conditions are compared to a single control group, statistical significance was tested using one-way analysis of variance (ANOVA), followed by Bonferroni’s multiple-comparison test. A *P* value of <0.05 was considered significant.

### Data availability.

Spatial transcriptomics data, including images, slide fiducials, filtered SpaceRanger outputs, and corresponding raw sequencing data are available via GEO accession GSE229699.
