# Supplementary figures and images for: Bacterial TLR2/6 Ligands Block Ciliogenesis, Derepress Hedgehog Signaling, and Expand the Neocortex
Source: mBio. 2023 Apr 13;14(3):e00510-23. doi: 10.1128/mbio.00510-23 (PMC10294647; doi:10.1128/mbio.00510-23)

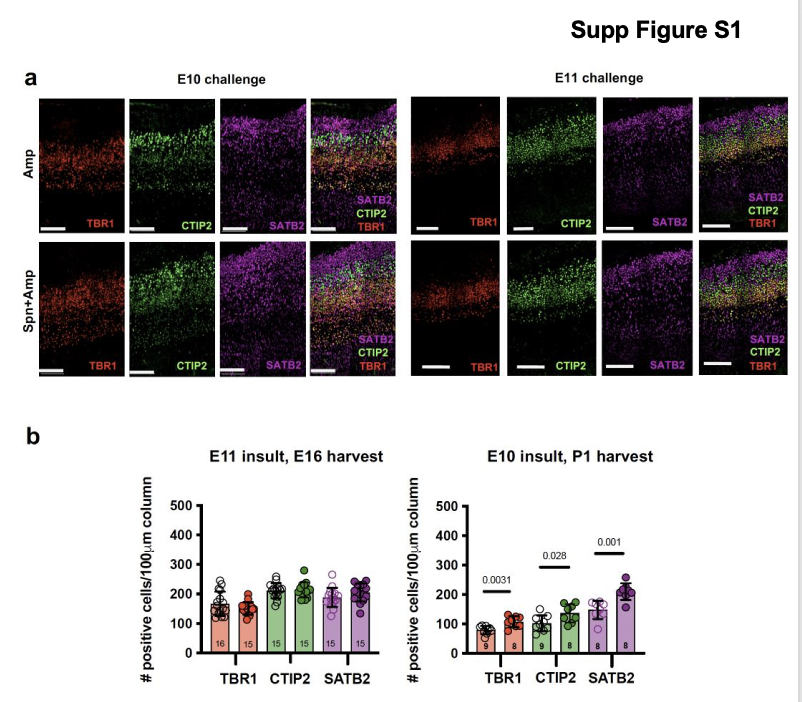

Supplement: FIG S1 [file mbio.00510-23-s0001.tif]

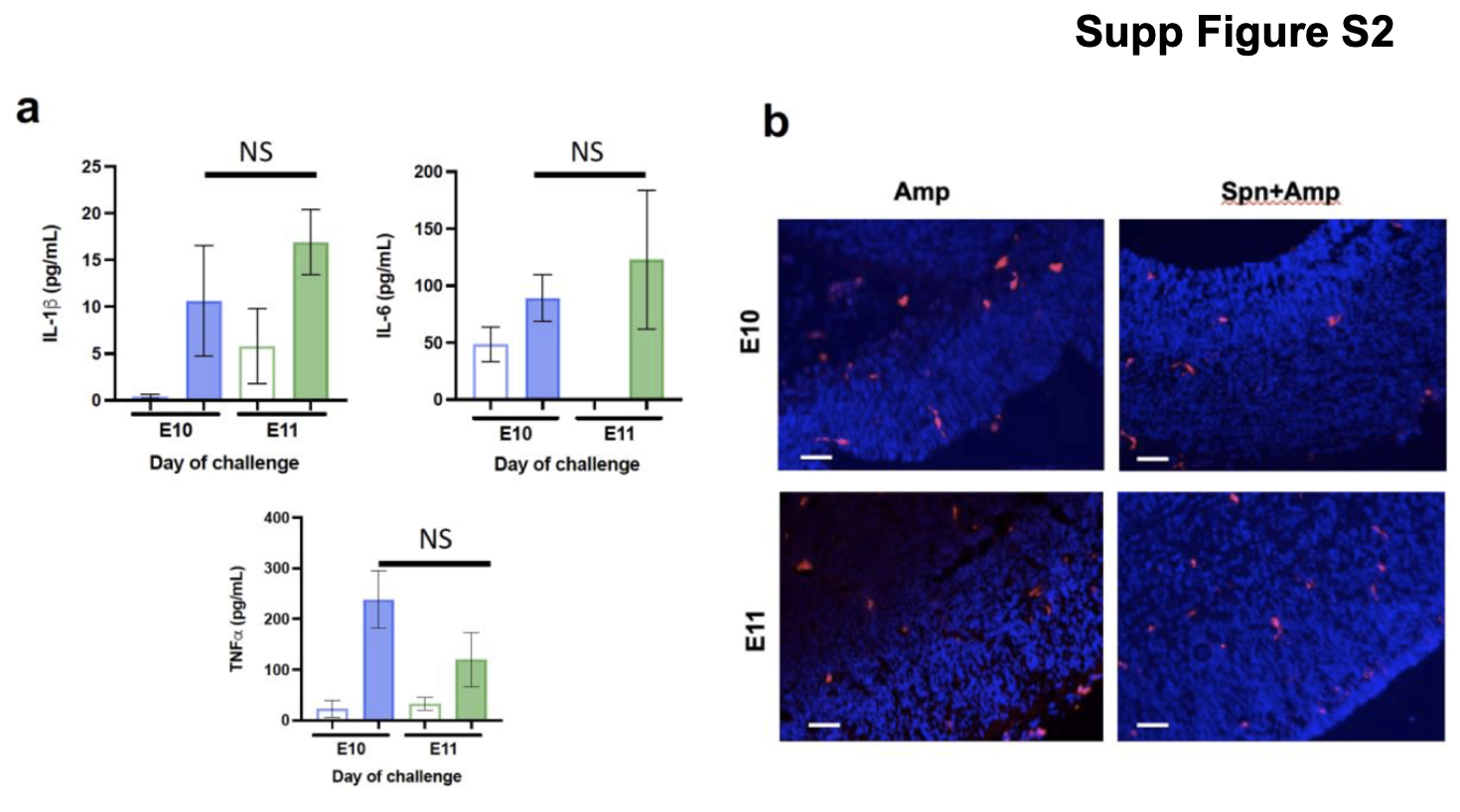

Supplement: FIG S2 [file mbio.00510-23-s0002.tif]

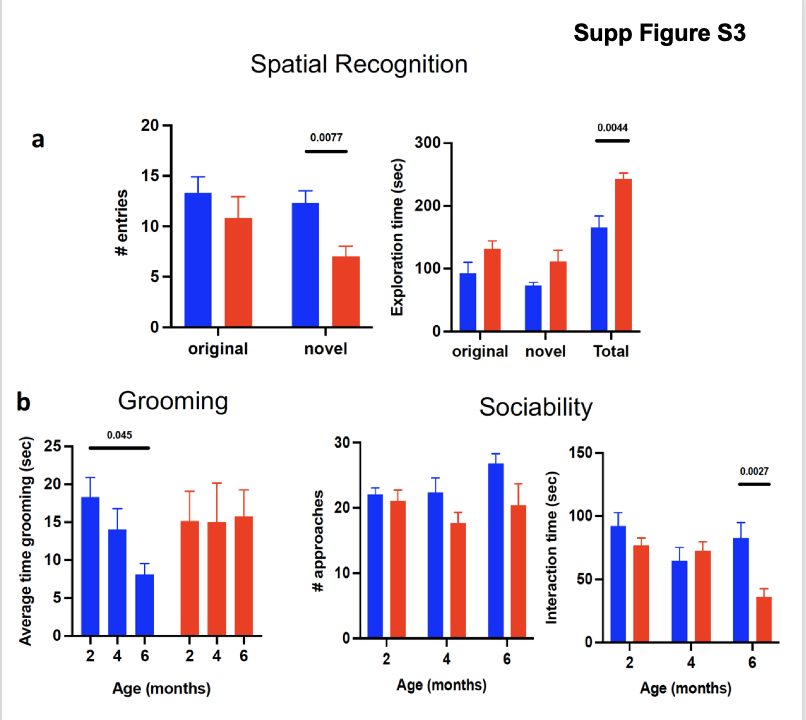

Supplement: FIG S3 [file mbio.00510-23-s0003.tif]

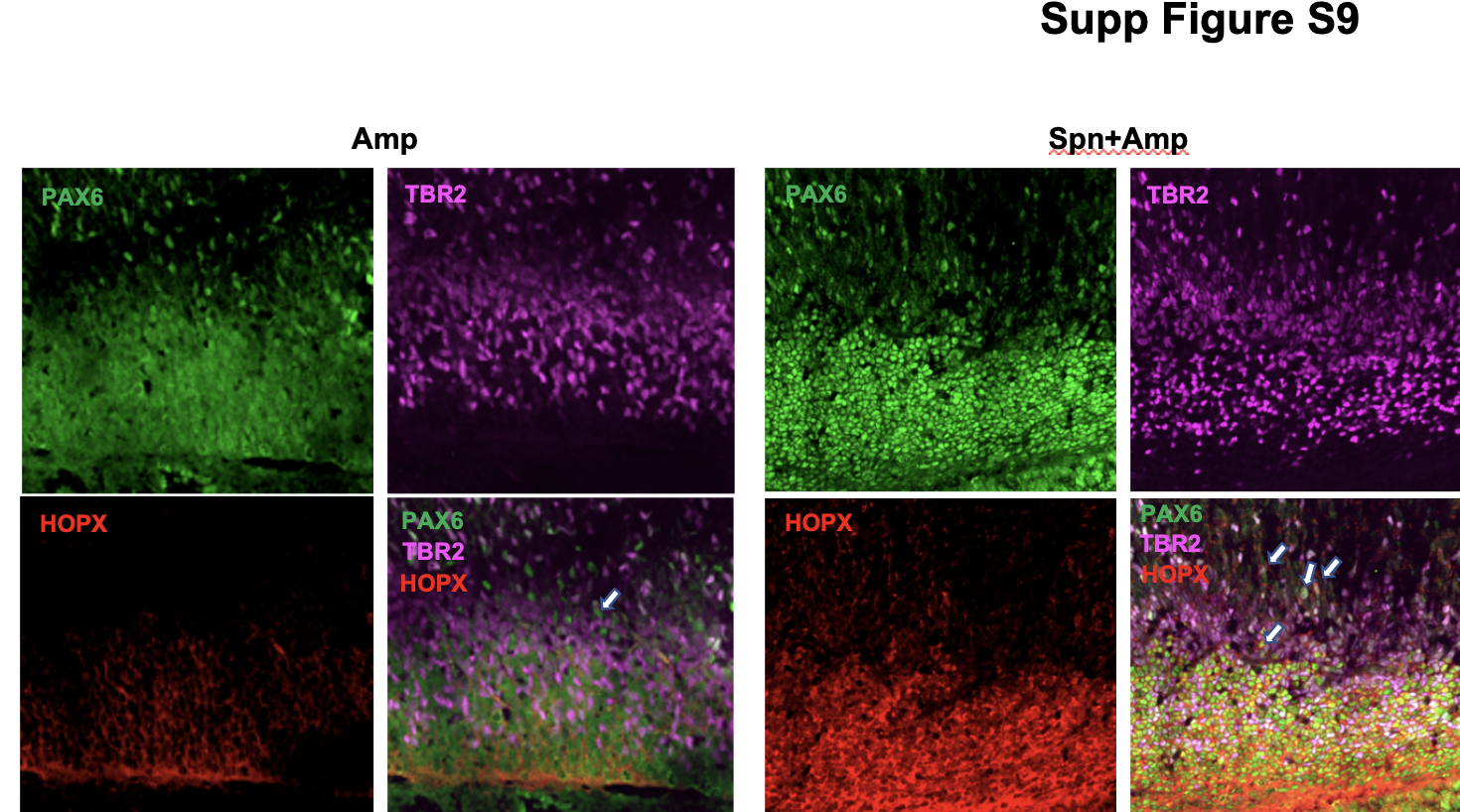

Supplement: FIG S9 [file mbio.00510-23-s0009.tif]

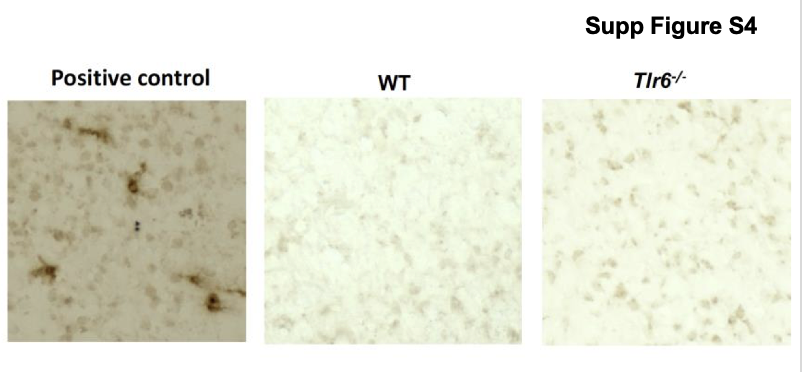

Supplement: FIG S4 [file mbio.00510-23-s0004.tif]

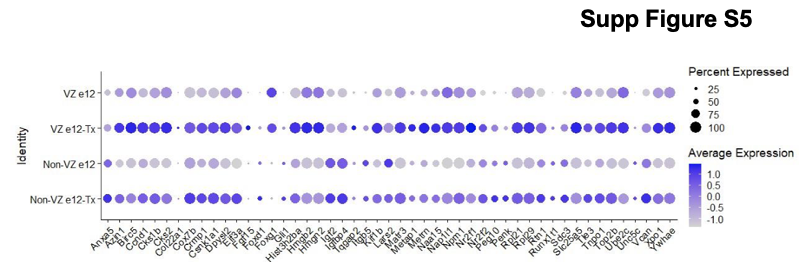

Supplement: FIG S5 [file mbio.00510-23-s0005.tif]

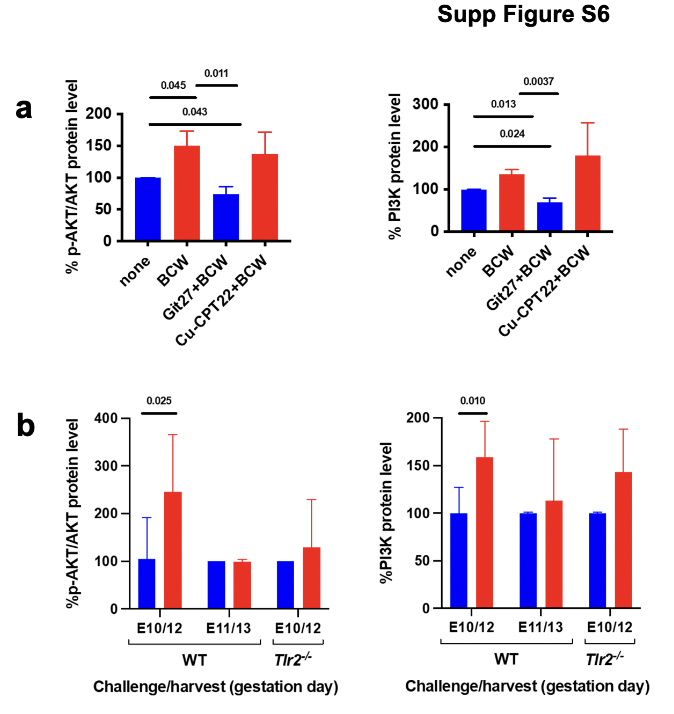

Supplement: FIG S6 [file mbio.00510-23-s0006.tif]

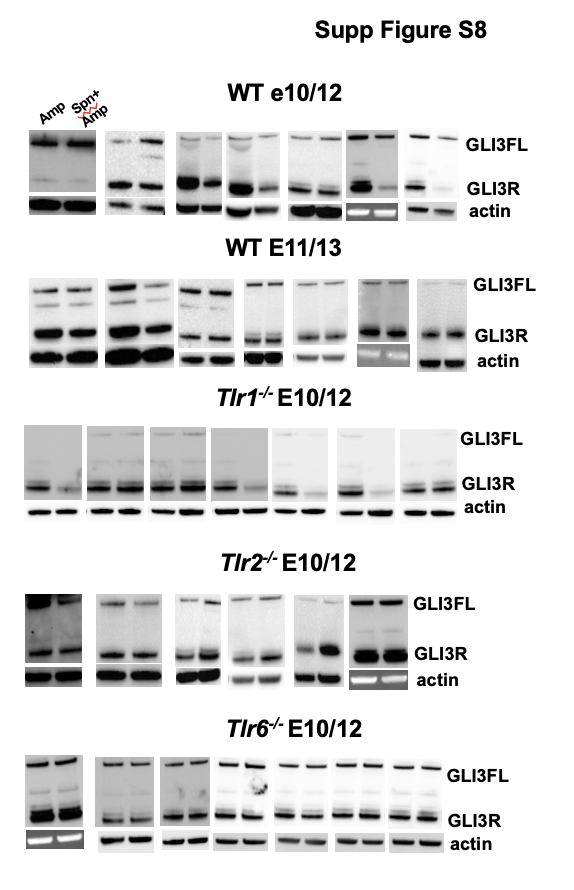

Supplement: FIG S8 [file mbio.00510-23-s0008.tif]

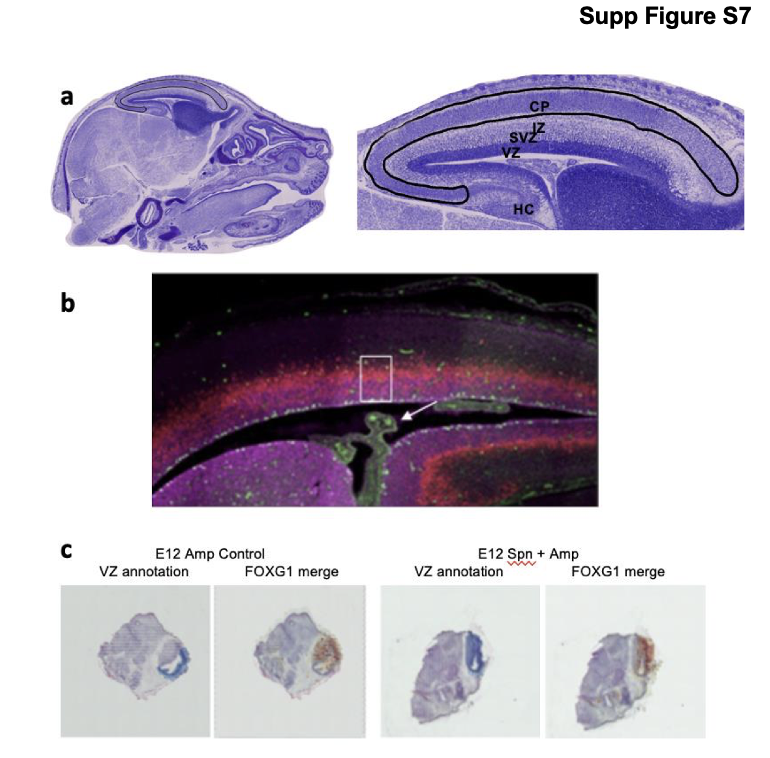

Supplement: FIG S7 [file mbio.00510-23-s0007.tif]
